# Mathematical Modeling for the Physiological and Clinical Investigation of Glucose Homeostasis and Diabetes

**DOI:** 10.3389/fphys.2020.575789

**Published:** 2020-11-25

**Authors:** Andrea Mari, Andrea Tura, Eleonora Grespan, Roberto Bizzotto

**Affiliations:** Institute of Neuroscience, National Research Council, Padua, Italy

**Keywords:** mathematical modeling, glucose metabolism, insulin secretion, glucose homeostasis, type 2 diabetes

## Abstract

Mathematical modeling in the field of glucose metabolism has a longstanding tradition. The use of models is motivated by several reasons. Models have been used for calculating parameters of physiological interest from experimental data indirectly, to provide an unambiguous quantitative representation of pathophysiological mechanisms, to determine indices of clinical usefulness from simple experimental tests. With the growing societal impact of type 2 diabetes, which involves the disturbance of the glucose homeostasis system, development and use of models in this area have increased. Following the approaches of physiological and clinical investigation, the focus of the models has spanned from representations of whole body processes to those of cells, i.e., from *in vivo* to *in vitro* research. Model-based approaches for linking *in vivo* to *in vitro* research have been proposed, as well as multiscale models merging the two areas. The success and impact of models has been variable. Two kinds of models have received remarkable interest: those widely used in clinical applications, e.g., for the assessment of insulin sensitivity and β-cell function and some models representing specific aspects of the glucose homeostasis system, which have become iconic for their efficacy in describing clearly and compactly key physiological processes, such as insulin secretion from the pancreatic β cells. Models are inevitably simplified and approximate representations of a physiological system. Key to their success is an appropriate balance between adherence to reality, comprehensibility, interpretative value and practical usefulness. This has been achieved with a variety of approaches. Although many models concerning the glucose homeostasis system have been proposed, research in this area still needs to address numerous issues and tackle new opportunities. The mathematical representation of the glucose homeostasis processes is only partial, also because some mechanisms are still only partially understood. For *in vitro* research, mathematical models still need to develop their potential. This review illustrates the problems, approaches and contribution of mathematical modeling to the physiological and clinical investigation of glucose homeostasis and diabetes, focusing on the most relevant and stimulating models.

## Introduction

Mathematical modeling in the field of glucose homeostasis has a time-honored tradition. One of the earliest “minimal” representations of the glucose-insulin system was published in the early 1960’s (Bolie, 1961). Before this study, glucose metabolism was intensively investigated by means of glucose tracers and using mathematical models. Steele et al. (1956) and Steele (1959) laid the foundations of glucose tracer modeling, describing a three-compartment model for the kinetics of labeled glucose and a single-compartment model for the assessment of glucose production in non-stationary conditions, which has become a standard for this purpose and is still in use. In the preceding years, mathematical theories for metabolic tracers flourished, e.g., in the Bulleting of Mathematical Biophysics, founded in 1939. The study of insulin secretion by modeling had a relatively more recent start, as the insulin radioimmunoassay was available only since the early 1960’s (Yalow and Berson, 1960). In the early 1970’s, Grodsky (1972) described a landmark model of the complex insulin secretion patterns that were observed in many *in vitro* and *in vivo* studies of early β-cell investigation. Since these early years, the use of mathematical models has been integrated in the mainstream of metabolic research. The growing worldwide concern on the societal impact of type 2 diabetes (T2D), a major disorder of glucose metabolism, has further stimulated metabolic research, including model-based studies.

In this article, we describe the ideas, problems, approaches, aims and achievements in the area of glucose homeostasis through various examples. The number of models in this area, from biochemical reactions in the cells to clinical trial simulation, is too large to allow a comprehensive review. A broad review is that by Ajmera et al. (2013), which discusses a wide class of models with different applications. Here, we have preferred to consider a selection of models that we repute influential in the physiological and clinical investigation of glucose homeostasis and T2D. Our intended audience includes scientists from both the mathematical and medical/biological fields, as this interdisciplinary area requires. With this review, we hope to enhance the reciprocal interest and trust of these two groups.

## Mathematical Modeling: Ideas, Aims, and Approaches

### Ideas and Aims

The natural idea underlying the use of mathematical models in physiology, as in other disciplines, is to employ the successful paradigm of physics for understanding, quantifying and predicting physiological processes. This paradigm, in its strict form, has some applications also in the study of metabolism. The mathematical theories of tracers have shown that relevant properties of the methodology can be demonstrated with rigorous methods (e.g., Norwich, 1977; Lassen and Perl, 1979). However, this is a rare case; most models involve considerable simplifications, which make their usefulness and success more challenging.

In physiological investigation, mathematical modeling has been focused on various aims. A humble, though relevant, aim has been the estimation of quantities that are not directly measurable. A famous old example is the so-called Fick’s principle to estimate cardiac output (Lassen and Perl, 1979), which rests on elementary equations for mass conservation and convective transport of a substance in a fluid. A similar need underlay the development of tracer methodologies, such as the cited Steele’s model (Steele, 1959), or the current standard model for calculating insulin secretion *in vivo* (Van Cauter et al., 1992). This category of models may embed assumptions that may be very solid, as in Fick’s principle, or not always appropriate, as for Steele’s model (Cobelli et al., 1987). This has led to progressive evolution of models for this purpose, as it has happened for Steele’s model (Radziuk et al., 1978; Mari et al., 2003).

An extension of this category of models includes widely used “calculators” of parameters of physiological interest that cannot be directly assessed or require complex experiments. A well-known example is the so-called “minimal model” (Bergman et al., 1979), an essential and approximate representation of the glucose-insulin homeostasis system aimed at quantifying insulin sensitivity, i.e., the ability of insulin to stimulate glucose utilization. Another famous example are the “HOMA” indices of insulin sensitivity and β-cell function, which originally were derived from a glucose homeostasis model (Matthews et al., 1985). The practical usefulness and relevance of these models has been crucial for their success.

An ambitious approach is to use models as unambiguous quantitative representations of particular physiological systems, aiming at elucidating the mechanisms underlying the observations. A good example is the cited model of insulin secretion by Grodsky (1972), which successfully described a series of complex experimental results using a consistent mathematical representation that provided hypotheses on the possible mechanisms determining the β-cell response. Another significant example is the model by Topp et al. (2000), which proposed an interesting mathematical formalization of the hypothesis that the decline of β-cell mass in T2D might be a consequence of damages to the β cells caused by hyperglycemia. This class of models is potentially of great interest, as it can provide insightful representations of complex physiological mechanisms, but it is also at high risk of speculations.

Complete representations of the glucose homeostatic system are an expected outcome of modeling in this field and indeed several representations of variable complexity have been proposed, as discussed below. However, glucose homeostasis simulators face the difficulty of dealing with a very complex system, the quantitative properties of which are still partially unknown. This makes the reliability of these simulators uncertain. Nevertheless, glucose homeostasis simulators have been proven helpful to test algorithms for optimal insulin administration regimens in type 1 diabetes, based on continuous glucose monitoring or the artificial pancreas (Hovorka et al., 2004; Dalla Man et al., 2007). A more ambitious model that extended its predictions beyond glucose homeostasis (e.g., to complications and pharmacoeconomics) has been the Archimedes model (Eddy and Schlessinger, 2003), which received considerable attention in the recent past.

The use of models to support development of drugs against T2D is a particular area. This approach, denoted as pharmacokinetic/pharmacodynamic (PKPD) modeling or pharmacometrics, and mainly aimed at providing advanced data analysis in the drug development process (e.g., Lee et al., 2011; Mould and Upton, 2012), is endorsed by drug regulatory agencies, such as the Food and Drug Administration. The typical PKPD approach is thus oriented toward model-based analysis of experimental data (i.e., it does not consider models for pure simulation) and employs population methods for the estimation of the model parameters (Mould and Upton, 2012). PKPD modeling methods have been used in the field of glucose homeostasis, with aims not necessarily related to drug testing. Models are mostly compartmental and often employ standard submodels of known properties (e.g., Landersdorfer and Jusko, 2008). While assessment of the model ability to describe the data adequately is a typical analysis step, less attention is paid to the adherence of the mathematical representation to the physiological knowledge.

### *In vitro* and *in vivo* Modeling

Since the early examples mentioned in the Introduction, models were developed to explain both *in vitro* and *in vivo* experimental data. *In vivo* models concerning glucose metabolism and insulin secretion have probably taken the lion’s share. More recently, however, following the great advancements of *in vitro* research, *in vitro* models, particularly for insulin secretion, have received remarkable attention, as discussed below. Notably, using modeling it is possible to interpret *in vitro* and *in vivo* experimental data with unifying mechanisms, when the same model, with appropriate parameter scaling, can predict both *in vitro* and *in vivo* data accurately. Because the methods of *in vitro* research are essential to understand the cellular mechanisms but cannot be applied to assess the relevance of the findings *in vivo*, mathematical modeling offers a way to bridge these two conditions. This approach has been for instance used for insulin secretion (Grespan et al., 2018), as discussed later.

### Strengths and Weaknesses of Mathematical Representations

The vast majority of models are based on ordinary differential equations, with some exceptions using partial differential equations (e.g., Salinari et al., 2011) or agent based modeling (Dehghany et al., 2015). The translation of the physiological mechanisms into a mathematical description has followed, however, different logics. In particular, the correspondence between physiology and equations has achieved a variable degree of neatness.

Some approaches attempt to condense the physiological complexity into model elements designed to capture the key physiological features, with a clear correspondence between the mathematical representation (e.g., the model parameters) and the physiology. One example is the cited insulin secretion model (Grodsky, 1972), in which, to describe accurately the dependence of first-phase insulin secretion on glucose levels, a characteristic hypothesis on the underlying process and a transparent mathematical description has been devised.

Other approaches are less concerned with the precise physiological significance of the equations, focusing on the ability of the model to describe the data adequately. This attitude typically accompanies the use of compartmental models, which can be easily configured and expanded to obtain a sufficient flexibility to describe the data. While this approach is not intrinsically flawed, it bears the risk of yielding models with an unclear or distorted relationship with the physiological system, as discussed below.

The mathematical representations are strengthened when they are supported by a formal analysis of their characteristics, which contributes to clarify the role of assumptions. This has been done occasionally for some models (e.g., Cobelli et al., 1987; Mari, 1997). For the minimal model, a formal analysis (Mari, 1997) has provided explanations for several issues encountered during its numerous applications.

### Success and Failure

Discussion of success and failure of models may be arbitrary and futile, as non-scientific aspects often influence these outcomes. Nevertheless, some remarks might be useful to improve reciprocal understanding between the experimental and modeling communities.

Perhaps the most successful models are those with a clear usefulness for various aspects of data analysis. The article describing the HOMA model (Matthews et al., 1985), which provides an easily computable index of insulin resistance, has received over 21,000 citations; the minimal model (Bergman et al., 1979), which has been used for insulin sensitivity assessment in may studies, over 1,300 citations; Steele’s model for tracer analysis (Steele, 1959) has been cited more than 1,800 times. A less striking but remarkable number of citations was received by several other models designed for data analysis, such as the model for calculating insulin secretion from C-peptide (Van Cauter et al., 1992) and the methods for assessing insulin sensitivity and β-cell function from an oral glucose tolerance test (Mari and Ferrannini, 2008; Cobelli et al., 2014). A message from these experiences is that models that provide relevant support to experimental data analysis are well received.

On the other hand, the success of models of remarkable mathematical sophistication and not oriented toward practical applications in experimental data analysis is of particular interest: it emphasizes the capacity of mathematical modeling to provide interesting interpretations of unexplained phenomena, which may be illuminating even beyond their adherence to the experimental data. Examples are the mentioned models of insulin secretion (Grodsky, 1972) and β-cell failure in T2D (Topp et al., 2000).

On the other side of the coin, indubitably some models may be an exercise with a weak rationale. Models with a poor relationship with experimental data and embedding questionable assumptions have a good reason for being ignored. Sometimes, however, modeling studies may be underappreciated due to the difficulty, inherent in the mathematical language, of communicating the fundamental results to a wider community. An important ingredient of success is thus to make the description of the model and its fundamental properties understandable to non-mathematical readers and to highlight the significance of the model in the research context.

## Physiological Background

### Glucose Homeostasis

This review focuses on models relevant for the study of glucose homeostasis, in which glucose metabolism and insulin secretion are the fundamental components. The glucose homeostatic system has received remarkable attention because its derangements lead to T2D, a pathology with a high societal burden.

The glucose homeostatic system is highly complex. Some mechanisms, such as glucose utilization in response to increasing insulin levels, have been widely studied and are quantitatively well characterized. Other aspects, such as the control of glucose production and the role of the central nervous system, are less clear. Poorly understood but very important aspects concern the relative role in the control of glucose levels of the various glucose homeostasis processes and the mechanisms underlying glucose tolerance deterioration that lead to overt T2D.

Glucose homeostasis is governed by an adaptive feedback control system. It includes some basic feedback loops and higher-level processes that affect the feedback loop characteristics to ensure tighter control. The adaptation capacity of the glucose homeostatic system is remarkable, as it can cope with wide variations of the subject’s conditions, such as obesity, starvation and pregnancy.

**FIGURE 1:**
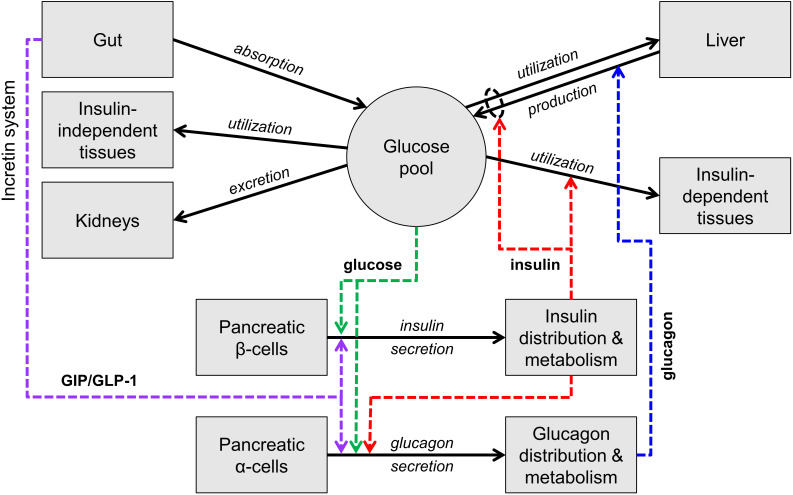
Scheme of the main mechanisms of glucose homeostasis. Mass fluxes are indicated as black solid arrows. Colored dashed arrows represent control signals (glucose or hormone concentrations) that regulate glucose fluxes or insulin and glucagon secretion. The scheme does not show adaptive control mechanisms (e.g., insulin secretion upregulation with insulin resistance).

Figure 1 is an essential representation of the glucose homeostatic system. Glucose levels are maintained by a balance of glucose delivery to the glucose pool (absorption from the intestine and production by the liver) and glucose utilization. Glucose utilization has an insulin-independent component (mainly in the central nervous system) and an insulin-dependent component (mainly liver, muscle, and adipose tissue). Glucose is excreted in the urine by the kidneys if glucose concentration is above a certain threshold. Glucose absorption from the intestine is not regulated by insulin, while hepatic glucose production and glucose utilization are directly controlled by insulin and, for production, by glucagon. Insulin and glucagon exert opposite actions: glucagon stimulates glucose production while insulin stimulates glucose utilization and inhibits glucose production.

Insulin and glucagon constitute the core feedback signals in the system. Glucose stimulates insulin secretion by the pancreatic β cells (and thus increases the insulin levels) and, together with insulin, suppresses glucagon secretion by the pancreatic α cells.

In normal life at fasting, the glucose level is maintained by balancing glucose production and utilization (mostly occurring in the central nervous system in this condition), through the contrasting actions of glucagon and insulin. When glucose decreases, insulin decreases and glucagon increases, leading to a compensatory increase in glucose production and decrease in glucose utilization. Opposite reactions take place when glucose increases.

With carbohydrate ingestion, glucose concentration increases due to glucose influx from the gut and the increase is counteracted by the same mechanisms described above. With nutrient ingestion, however, another mechanism contributes to glucose lowering, the so-called entero-insular axis or incretin system. The passage of nutrients in the intestine stimulates the secretion of two hormones produced by specific intestinal cells, denoted as GIP and GLP-1 or incretin hormones, which potentiate insulin secretion. GLP-1 also inhibits glucagon secretion, thus further suppressing glucose production.

While these multiple feedback loops control glucose concentration during fasting and feeding in normal conditions, the adaptive mechanisms take place when the conditions are altered, such as with weight gain. Weight gain typically causes insulin resistance, i.e., hampers the ability of insulin to enhance glucose utilization and suppress glucose production. If insulin resistance were not compensated, glucose concentration would increase above the normal levels. The main adaptive mechanism activated with insulin resistance involves insulin secretion, which is upregulated, i.e., for the same glucose concentration the β cells secrete more insulin.

### Derangements of Glucose Homeostasis

Various mechanisms may be malfunctioning and cause an increase in glucose concentration, a condition denoted as glucose intolerance. The mechanisms underlying glucose intolerance have been widely studied, and two factors have been highlighted as crucial: insulin resistance, described previously, and β-cell dysfunction, i.e., the inability of the β cells to secrete appropriate amounts of insulin. In T2D, insulin resistance is present and β-cell function is compromised and often declines as the disease progresses. Understanding and preventing the progressive β-cell function deterioration is a crucial issue to prevent T2D worsening, and an area for modeling.

## Overview of Models

In the following sections, on a historical background we discuss several models proposed to describe various aspects of the glucose homeostasis system and to address specific problems. Models are grouped according to the glucose homeostasis subsystem they describe.

### Glucose Utilization and Insulin Action

The study of glucose kinetics with tracers has been among the first applications of modeling. The whole-body tracer methods initially relied on the analysis of the tracer concentration curves in blood after a bolus injection, which were fitted with a sum of exponentials to calculate glucose clearance and distribution volume in the body. This approach naturally led to the use of compartmental models (e.g., Steele et al., 1956, in dogs), which showed the existence of multiple compartments. Since the early studies, multi-compartment models of glucose kinetics have thus become a sort of standard. Based on this approach, the effects of insulin on glucose utilization have been described by Insel et al. (1975) using a model that represents the progenitor of many successive models, still in use today. Insel’s model couples a three-compartment model of insulin kinetics (Sherwin et al., 1974) with a three-compartment model of glucose kinetics. The three-compartment models featured a “central” compartment, corresponding to the plasma pool (where glucose, tracer and insulin are measured) and two “peripheral” compartments. The model has introduced the important concept that the action of insulin on glucose utilization is delayed compared to the insulin concentration profile in plasma. In the multi-compartment model, this was achieved by including a glucose utilization rate from a peripheral glucose compartment controlled by insulin concentration in a peripheral insulin compartment, sometimes denoted the “remote” (with respect to plasma) insulin compartment.

While Insel’s model laid the foundations of successive models describing glucose-insulin interactions, compartmental models were widely used to calculate glucose fluxes (utilization, production, and absorption) from the analysis of tracer data in non-steady state conditions, such as after insulin infusion or an oral glucose load. After the widely used Steele’s single-compartment model (Steele, 1959), Radziuk et al. (1978) proposed a more accurate approach based on a two-compartment model. These models did not assume a structural dependence of glucose utilization on insulin concentration, as in Insel’s approach; they were used to calculate the time course of glucose utilization (in addition to glucose appearance from oral ingestion and hepatic glucose production) from glucose and tracer concentrations. This generality allowed wide use of the models in many studies.

Compartmental models have been used for the study of glucose kinetics beyond their limitations, highlighted in authoritative commentaries (e.g., Zierler, 1981). Insel’s model already embedded the questionable principle that compartments could be identified with physiological entities. A typical example is the so-called plasma compartment, which is associated to measurements performed in plasma, such as glucose and insulin concentrations. This association bears the unrealistic expectation that the estimate of the compartment volume, obtained from the extrapolation to zero of, e.g., a tracer disappearance curve, corresponds to the plasma volume. Detailed recordings of the concentration profiles on the time scale of seconds are not compatible with this view (e.g., Avram et al., 1997).

An alternative to compartmental models are those embedding a description of the circulatory loop (arteries – tissues – vena cava – heart/lungs – arteries) coupled with the classical methods for the study of organ kinetics (Lassen and Perl, 1979), used to represent the lumped organs. This approach, conceived long ago (Waterhouse and Keilson, 1972), has the advantage of employing mathematical representations that are based on solid physical principles (convective transport and mass conservation) and have well characterized properties (Mari, 1993, 1995a,b). This class of models, sometimes denoted as “circulatory models,” may include finite delays in convective transport, observed in real data (e.g., Avram et al., 1997), or responses that are not multi-exponential. The theory of circulatory models has provided rigorous answers to the problem of the determination of the distribution volume, showing that a fraction of this volume is not determinable from classical kinetics experiments (Mari, 1993). The expectation of compartmental models to overcome this indeterminacy based on model configurations inspired to a correspondence between compartment and organs (e.g., Cobelli et al., 1984; Ferrannini et al., 1985) has no solid foundations and provides arbitrary results (Mari, 1993). Circulatory models provide a framework to describe whole-body glucose kinetics using mathematical representations that bear a close relationship with the physiological system, as done for the analysis of non-steady state tracer data (Mari et al., 2003) or for insulin action (Bizzotto et al., 2016).

The study of glucose utilization is linked to the quantification of insulin sensitivity, i.e., the ability of insulin to stimulate glucose utilization and suppress glucose production. This problem was addressed by Insel’s model, which was based on experimental data from a euglycemic glucose clamp, the reference test for the assessment of insulin sensitivity (Ferrannini and Mari, 1998). The notion that the effects of insulin on glucose utilization could be well represented by modeling, let to the proposal of using a model to estimate insulin sensitivity from a test simpler than the euglycemic glucose clamp. The proposed model (Bergman et al., 1979), which was successively denoted as the “minimal model” (e.g., Bergman et al., 1981) alluding to its essentiality, can be considered a simplification of Insel’s model, applied to an intravenous glucose tolerance test (IVGTT), i.e., a bolus injection of glucose. The minimal model inherited from Insel’s model the notion that the effects of insulin are delayed with respect to plasma insulin concentration (the remote insulin compartment) and used a single compartment simplification for glucose kinetics. The important advantage of the simplification was the possibility of estimating the model parameters, and in particular an insulin sensitivity index, from a clinically usable test such as the IVGTT. This was the basis for the widespread use of the minimal model.

For various reasons the minimal model has attracted great attention, both from the modeling and the experimental community, becoming an iconic model. Initially, some studies evaluated the degree of concordance between the insulin sensitivity estimates from the minimal model and the reference method of the glucose clamp (e.g., Finegood et al., 1984; Saad et al., 1994). Later, criticism has been raised regarding the excessive simplification of the model [e.g., Caumo et al., 1996 and the discussion on the IVGTT models by Palumbo et al. (2013)], which became evident in the difficulty of data fitting and negative (or zero) estimates of the insulin sensitivity index (e.g., Saad et al., 1994; Godsland and Walton, 2001). A two-compartment evolution of the minimal model has been proposed to overcome these problems (Vicini et al., 1997; Cobelli et al., 1999), as well as other model formulations (Palumbo et al., 2013). In spite of its limitations, the minimal model remains a valuable approach for the assessment of insulin sensitivity, as discussed in our formal analysis of the role of the minimal model assumptions (Mari, 1997). However, while experimental studies with the minimal model have certainly expanded our knowledge on glucose homeostasis and have probably increased the acceptance of mathematical models among experimentalists, the minimal model *per se* has not improved much our mechanistic understanding of the relationships between insulin and glucose utilization, beyond its original foundations by Insel. In the early 2000’s, the idea of using a model to assess insulin sensitivity from a clinically applicable test, underlying the minimal model, has been extended to the oral glucose tolerance test (OGTT) or a meal test, for which two model-based indices have been proposed (Caumo et al., 2000; Mari et al., 2001).

Advancements over Insel’s description of insulin action on glucose utilization have been made by some models that attempt to describe two phenomena known since long time: the non-linear dependence of glucose utilization on insulin concentration (e.g., Rizza et al., 1981) and on glucose concentration (e.g., Best et al., 1981). Because the reasons underlying these findings are still unclear and data to support mathematical descriptions are scarce, these phenomena are often not represented in the models of insulin action (e.g., Jauslin et al., 2011) or the representations are conceptually rational but not directly tested against experimental data (e.g., Dalla Man et al., 2007). We have addressed these problems using a circulatory model, and showing that at constant glucose concentration a Michaelis–Menten relationship between insulin concentration and glucose utilization was in good agreement with the experimental data (Natali et al., 2000). To represent the non-linear dependence of glucose utilization on glucose concentration, we have later used a similar model for the relationship between insulin concentration and glucose utilization, combined with a Michaelis–Menten equation expressing glucose utilization as function of glucose concentration (Bizzotto et al., 2016). This model was able to describe several tracer-based studies with a wide range of glucose and insulin concentrations and has highlighted that the non-linear dependence of glucose utilization on glucose concentration is a quantitatively relevant phenomenon in glucose homeostasis.

### Insulin Secretion

#### Assessment of Insulin Secretion

Early investigation on insulin secretion from pancreatic β cells, both *in vitro* and *in vivo* [e.g., the landmark studies by Grodsky (1972) in the perfused pancreas and by Cerasi et al. (1973) in humans], has been based on the measurement of insulin concentration. While *in vitro* research still uses insulin concentration, studies *in vivo* have found that insulin, secreted by the pancreas in the portal vein, is massively removed (by ∼50%) as it crosses the liver (see the section on insulin clearance below). As hepatic insulin extraction may be variable, both within and between subjects, plasma insulin concentration is not reputed to represent insulin secretion accurately. For this reason, an alternative methodology has been devised, based on the measurement of plasma C-peptide, a molecule secreted with insulin in equimolar amounts and not substantially removed by the liver (Eaton et al., 1980). Because of the linearity of C-peptide kinetics, insulin secretion can be calculated from C-peptide concentration using a C-peptide model and deconvolution approaches (Eaton et al., 1980; Hovorka and Jones, 1994). The use of this method in experimental studies would require the individual assessment of the C-peptide model parameters. To overcome this complication, a standardized C-peptide model has been proposed by Van Cauter et al. (1992), in which the model parameters are individualized based on anthropometric data. This model has become the standard method for calculating insulin secretion *in vivo*. In practice, no alternative exists, also because obtaining synthetic C-peptide to assess C-peptide kinetics is difficult. Regretfully, Van Cauter’s model has not been developed for all the situations to which it has been applied. The dataset on which it was based included lean and obese subjects with normal glucose tolerance and T2D, but not children, pregnant women, severely obese subjects and type 1 diabetic patients, populations in which the model has nevertheless been used. Furthermore, in Van Cauter’s model, the dependence of the C-peptide kinetic parameters, and in particular of C-peptide clearance, on the subject characteristics embeds thresholds (on the body mass index and glucose tolerance) and is thus not continuous. This may be problematic in longitudinal studies, as it may generate spurious sudden changes in C-peptide clearance when the thresholds are crossed.

#### Clinical Models for β-Cell Function Assessment

The early models for the assessment of β-cell function from clinical tests, such as the IVGTT, were developed in parallel with the minimal model (Toffolo et al., 1980; Bergman et al., 1981). These models, still based on insulin concentration, used a very simplified representation of the β-cell response to an IVGTT. The biphasic insulin secretion response during this test was quantified with a first-phase index, expressing the expected contribution of the initial insulin secretion burst to insulin concentration, and a second-phase index, obtained from an empirical model relating insulin secretion to glucose concentration and time. This empirical model has been employed in several studies, but the relationship of the estimated first- and second-phase parameters with the classical indices from the hyperglycemic clamp have not been assessed, to our knowledge.

Between the late 1990s and the early 2000s, renewed attention has been paid to the OGTT as an effective clinical test to assess β-cell function, besides insulin sensitivity, using empirical formulas and model-based approaches. Four relevant models were conceived in this period to assess β-cell function and are still in use (Hovorka et al., 1998; Breda et al., 2001; Cretti et al., 2001; Mari et al., 2002). OGTT-based methods are of considerable interest in clinical research because of the relative simplicity of the test, its diagnostic value for glucose tolerance and the possibility to assess both insulin sensitivity and β-cell function. In addition, the OGTT represents a physiological perturbation of the glucose homeostatic system, compared to the IVGTT or the glucose clamps, and, more importantly, it involves the incretin system, which is a relevant player in insulin secretion, as discussed below.

The cited models share some principles (all rely on C-peptide for insulin secretion assessment) but also have important differences. The earliest model by Hovorka et al. (1998) is based on a simple linear dose-response relating insulin secretion to glucose concentration. The model by Cretti et al. (2001) is in essence an extension of Hovorka’s model that includes a delay between the changes in glucose concentration and those of insulin secretion. The original model parameters are, however, transformed according to an empirical logic to obtain a β-cell function index. The other two models (Breda et al., 2001; Mari et al., 2002) include a representation of early insulin secretion, which is reputed to be a manifestation of the mechanisms underlying first-phase secretion. Early insulin secretion is modeled as a function of the derivative of glucose concentration (the “dynamic” or “derivative” insulin secretion component), which is positive when glucose concentration increases and zero otherwise. This representation followed an elaboration by Ličko (1973) of Grodsky’s model of first-phase insulin secretion (Grodsky, 1972). The two models, however, differ in the description of sustained secretion during the OGTT. Breda’s model (Breda et al., 2001) included a first-order delay between the changes in glucose concentration and insulin secretion. This mechanism explained the observation that, in the late phase of the OGTT, insulin secretion is higher, for the prevailing glucose level, compared to baseline. Our model (Mari et al., 2002) did not represent this phenomenon as a delay, but as a time-dependent potentiation, according to the studies by Cerasi et al. (1974). We avoided the delay model also because in the perfused pancreas the insulin secretion response to a square wave of hyperglycemia is incompatible with a first-order delay, as it shows a slow onset but a fast offset (Bergman and Urquhart, 1971; Grodsky, 1972). Although these differences are important for the interpretation of insulin secretion during an OGTT (Mari and Ferrannini, 2008), all these models have shown their usefulness for the assessment of β-cell function in a large number of studies.

#### β-Cell Models

Numerous models have been proposed to explain the complex insulin secretion patterns observed *in vitro* and investigate the underlying mechanisms. Table 1 summarizes the β-cell models reviewed here and highlights some of their relevant features, discussed in detail in the text.

**TABLE 1:** Summary of the β-cell models.

Study	Mechanisms^1^		Data^2^		Explanation of T2D^3^
	Calcium	Glucose	*In vitro*	*In vivo*	
Grodsky, 1972	No	Explicit	++	∅	No
Cerasi et al., 1974	No	Explicit	∅	+	Yes
Overgaard et al., 2006	No	Explicit	∅	+	(Yes)
Giugliano et al., 2000	Explicit	Explicit	−	∅	Hypothesis
Bertuzzi et al., 2007	Implicit	Explicit	+	+	Hypothesis
Chen et al., 2008	Explicit	Implicit	+	∅	Hypothesis
Pedersen and Sherman, 2009	Explicit	Implicit	−	∅	No
Stamper and Wang, 2013	No	Explicit	−	∅	No
Dehghany et al., 2015	Implicit	Explicit	+	∅	No
Palumbo and De Gaetano, 2010 and De Gaetano et al., 2015	No	Explicit	−	−	Hypothesis
Grespan et al., 2018	Explicit	Explicit	++	++	Yes
Pedersen et al., 2019	Explicit	Explicit	++	∅	Hypothesis

*^1^Explicit or implicit mathematical representation of the role of calcium and glucose on insulin secretion. Only explicit representations allow simulations with arbitrary calcium or glucose data. ^2^Simulations with direct comparison with experimental in vitro or in vivo data: not simulated (∅); no comparison (−); limited comparison (+); extensive comparison (++). Studies with no direct comparison show simulations only, without experimental data. ^3^Analysis of the mechanisms underlying type 2 diabetes: not considered (No); hypotheses provided without comparison with real data (Hypothesis); hypotheses based on the comparison with real data (Yes). The study by Overgaard et al. (2006) includes data from relatives of type 2 diabetic patients who developed diabetes.*

Grodsky’s model (Grodsky, 1972) explained the biphasic insulin secretion pattern observed after a brisk elevation in glucose concentration with distinct mechanisms for the initial short secretion peak and the slowly ascending second insulin secretion phase. First-phase secretion was described with the original hypothesis that insulin in the β cells is contained in “packets” that are quickly released from a “labile pool” when glucose concentration exceeds a packet-specific threshold. This model was able to reproduce accurately the sharp first-phase insulin secretion peaks and their dependence on the glucose concentration increase with a suitable distribution of the packet thresholds. Second-phase insulin secretion was described as a process of refilling of the labile pool from a stable compartment. This representation was able to reproduce data from several tests, most importantly the response to a square wave of hyperglycemia followed, after a short time, by a second hyperglycemic step. When Grodsky’s model was proposed, the knowledge of the cellular mechanisms underlying insulin secretion was quite rudimentary. That the distribution of the packet thresholds could be related to insulin granule heterogeneity was a hypothesis, as it was the possibility that the heterogeneity could concern β cells. Interestingly, however, the labile pool and refilling are concepts that are still in use in the current research on β-cell physiology and modeling, as discussed below. In addition, the idea of a distribution of heterogeneous β-cell characteristics is still under discussion.

Among the historical models of the insulin secretion mechanisms, Cerasi’s model (Cerasi et al., 1974) is noteworthy at it interprets the biphasic insulin secretion response to a hyperglycemic step differently from the prevalent pool-based view. This model described first-phase secretion as the effect of an initiation phase, rapidly followed by an inhibition (refractory) phase. The ascending second phase and the amplification of the secretory response with two successive glucose stimuli was ascribed to potentiation mechanisms, observed experimentally. The model was used to analyze experimental data, including subjects with T2D. The reduced secretory response in T2D was attributed to reduced initiation or increased inhibition.

The model by Overgaard et al. (2006) is a simplification of Grodsky’s model and describes two insulin pools (denoted as “active” and “passive”), which shape the dynamic secretory response, and a glucose-dependent pool refilling (denoted as “provision” as in Grodsky’s terminology), which determines sustained insulin secretion. Grodsky’s concept of a distribution of the packet thresholds is reformulated using a function that increases with glucose concentration, augments the active pool size and sustains first-phase insulin secretion. First-phase insulin secretion is also governed by a term that is dependent on the derivative of glucose concentration. In the OGTT, the refilling includes an incretin effect component, as discussed in the specific section below. The model was conceived to estimate clinically meaningful β-cell function parameters from an IVGTT or OGTT. However, the clinical value of the parameters and the potential advantages of this more elaborate model with respect to the simpler approaches described above have not been demonstrated. The study included a group of relatives of T2D patients that subsequently developed overt diabetes, from which the defects underlying T2D was hypothesized. The alterations found in this group mainly concerned the redistribution rate constant from passive to active packets and the threshold distribution function.

Although the role of calcium in insulin secretion was already known when these early models were proposed, the underlying mechanisms were elucidated considerably later (see e.g., Henquin, 2000 for a summary). An early finding was the link between calcium and membrane electrical activity, modulated by glucose, which stimulated the development of models of membrane potential oscillations since the early 1980s (Chay and Keizer, 1983). In this area, several models have been proposed to represent the complex interplay between glucose metabolism, membrane ion channels and potential, and calcium levels, as reviewed in (Ajmera et al., 2013; Felix-Martinez and Godinez-Fernandez, 2014; Bertram et al., 2018).

Calcium dynamics started to be incorporated in models of insulin secretion since the work by Giugliano et al. (2000). The model represented the metabolic pathways related to glucose transport across the cell membrane, ATP production following glucose metabolism and the electrophysiological events that follow intracellular glucose increase. The electrophysiological model predicted the changes in membrane potential in relation to the potassium, sodium and calcium currents. The intracellular calcium dynamics modulating calcium and sodium currents was described by a two-compartment model derived from Chay (1996). The insulin granules were represented in one of the following three states: “recovered” (ready-releasable), “active” (during the fusion to the plasma membrane) and “inactive” (granules in the reserve pool). Readily releasable granules are released when calcium spikes occur, in quantities that depend on both the amount of activated anchoring proteins (calcium dependent) and the number of readily releasable granules. These mechanisms determine first-phase insulin secretion. The second phase depends on the recovery and mobilization of the granules to the readily releasable pool, which is represented as a first-order process occurring over a fixed time scale. Thus, the refilling rate of the ready releasable granules does not depend on glucose concentration, as it would be expected from experimental data. The model represented β-cell dysfunction in T2D as a desensitization of ATP-sensitive potassium channels to intracellular ATP concentration. All simulations qualitatively represented insulin secretion without a direct comparison with experimental data.

The study by Bertuzzi et al. (2007) gave a representation of insulin granule trafficking that implied a role of cytoplasmic calcium in exocytosis, but calcium was not explicitly modeled. Both the biochemical events that lead to changes in ATP concentration and cytoplasmic calcium concentration in response to a glucose stimulus are represented as time changes of rate coefficients regarded as “control” signals that regulate granule trafficking. The model includes a reserve granule pool and pools of docked, immediately releasable and fused granules. First-phase insulin secretion mainly depends on the insulin amount in the pool of immediately releasable granules and on the product of functions representing ATP sensitivity to glucose and calcium sensitivity to ATP. Second-phase insulin secretion is regulated by the size of the reserve pool and the rate coefficient of granule translocation and priming, related to the ATP-to-ADP ratio. The model does not include explicitly the regulatory role of calcium in granule translocation and thus in the second-phase insulin release, shown in several studies. The model was able to reproduce qualitatively insulin secretion in some classical *in vitro* studies, although without a direct comparison with experimental data, and data from a hyperglycemic clamp *in vivo*. The possible reasons underlying defective insulin secretion in T2D were not deeply investigated, but it was suggested that the translocation from the reserve pool to the docked granules, granule fusion and the release of insulin from fused granules could be involved.

Chen et al. (2008) developed a model representing the exocytosis cascade (composed of docked, primed, readily releasable, and fused granules) and a two-compartment model of intracellular calcium dynamics. Both L-type and R-type voltage-sensitive calcium channels control calcium influx when the cell is depolarized and four types of calcium transporters regulate its clearance from the cytosol. The space surrounding L-type channels is one of the calcium compartments, denoted as “microdomain”; in this region, in which calcium concentration can reach 20–30 μmol/L, insulin granule exocytosis takes place. The second calcium compartment represents cytosolic calcium, where concentration is in the order of 200–300 nmol/L during cell depolarization. The dependence of calcium concentration on the glucose stimulus is not described but calcium follows membrane depolarization, which is represented as a square wave. First-phase insulin secretion depends on the size of the readily releasable pool of granules, which is mainly determined by the granule priming rate. Second-phase secretion is dependent on the resupply rate of granules from the reserve to the docked pool. The dependence of the priming rate and the refilling rate on glucose is not modeled, but changes are empirically determined to reproduce the simulated data. The model reproduces several experimental conditions such as a hyperglycemic clamp, a glucose ramp and the response to two consecutive hyperglycemic glucose steps separated by a period of rest, and investigates the role of R and L-type calcium channels. However, the model-predicted cytosolic calcium concentration and insulin secretion are not compared to real data. In addition, the relationships between glucose and membrane depolarization controlling calcium and the refilling rate are not mathematically represented but set empirically.

Pedersen and Sherman (2009) have extended Chen’s model by including a pool of granules with a higher calcium sensitivity and responding to cytosolic calcium rather than to calcium in the microdomain, which triggers exocytosis of the immediately releasable pool. Insulin granules activated in this way, called newcomers, can be secreted without passing through the docked status, away from the microdomain. The model ascribes to these granules the main role during second-phase insulin secretion. First-phase insulin secretion depends on the granules close to the L-type calcium channels, in the microdomain, as in Chen’s model. The model investigates the role of R and L-type calcium channels, Syntaxin-1A protein and of the low and high sensitivity granule pools on biphasic insulin secretion, reproducing the secretory response to a hyperglycemic clamp in normal conditions and with knockdown of the R-type or L-type channels or Syntaxin-1A. As for Chen’s model, however, the described mechanisms are not explicitly linked to glucose concentration and simulations are not compared to the experimental data. In contrast to Chen’s model, this model does not describe the effect of calcium on granule mobilization.

The model of Stamper and Wang (2013) represents granules in a docked, primed and fused status, a reserve pool and a pool of newcomer granules. The translocation rates among pools are glucose dependent and the model suggests that insulin granule mobilization from the reserve pool is a critical factor for second-phase insulin secretion. First-phase insulin secretion depends on the rapid depletion of the primed, readily releasable granules. The model reproduces insulin secretion in response to several classical tests, although simulations are not directly compared with real data. The model does not describe the role of calcium on insulin secretion explicitly.

The model by Dehghany et al. (2015) is the first and only agent-based space-resolved model for insulin granules dynamics in pancreatic β-cells. The model includes a spatial description of the β cell including insulin granules, microtubules and actin filaments and simulates the movement of the secretory granules from the inner cell to the plasma membrane, regulated by glucose levels. The model represents docked, primed and newcomer granules. Docked granules can be released if they are close enough to calcium channels and a large part of these granules undergoes exocytosis after priming upon glucose stimulation: these granules constitute the readily releasable pool. Exocytosis depends on the number of readily releasable granules and on the number of opened calcium channels. A primed granule fuses with the plasma membrane when at least one of the calcium channels of the hosting docking site is open. The model does not consider ion currents explicitly but uses an empirical function to determine the opening probability of the channels. Exocytosis takes place with a certain delay. First-phase insulin secretion depends on the size of the readily releasable pool, with the time and shape of the peak modulated also by the exocytosis latency. Second-phase insulin secretion depends mainly on newcomers granules, and the rising second phase is modulated by an increased number of docking sites and a decreased priming rate upon glucose stimulation. The model reproduces and compares with experimental data several experimental tests: the response to continuous and intermittent membrane depolarization with and without glucose, a hyperglycemic clamp and two successive hyperglycemic steps followed by a period of rest.

The models by Palumbo and De Gaetano (2010) and De Gaetano et al. (2015) resume Grodsky’s concept of the distribution of thresholds in glucose sensing and, in some way, Cerasi’s proposal of a refractory state after rapid insulin release and potentiation with hyperglycemia. Secretory units, identified with islets, are supposed to release an insulin packet when a given glucose threshold is crossed and then enter a refractory state (i.e., a sudden increase in the threshold) that progressively returns to the normal state. The size of the packet is modulated by glucose, which acts with a delay. The model parameters are assumed to follow assigned distributions (typically lognormal) in one million of simulated units. The second study used a more complex representation of packet size modulation by glucose and a modified distribution of the glucose thresholds. Both studies also include a glucose-insulin model to simulate some experimental conditions *in vivo*. The studies have specifically addressed the problem of insulin secretion oscillations, but the second study simulated also more classical tests, such as those considered by Grodsky and an IVGTT in subjects with normal glucose tolerance and T2D. In T2D, a reduction of the potentiating effect of glucose, of the average basal packet size and possibly of the glucose threshold distribution was postulated, but the simulated IVGTT was not compared with real data.

We recently envisaged a model with which *in vitro* and *in vivo* data could be interpreted in a unifying framework, aiming at elucidating the possible cellular defects underlying β-cell dysfunction in T2D (Grespan et al., 2018). We based the mathematical representation of the cellular events on the current description of the fundamental mechanisms of insulin secretion (Henquin, 2000), similarly to several models described above. The core of the model is an immediately releasable pool of insulin granules, the content of which depends on calcium-mediated exocytosis (the “triggering pathway”), and on a calcium- and glucose- mediated refilling flux (the “amplifying pathway”). These two processes are represented with functions of calcium and glucose. Calcium is determined from glucose using a dose-response function derived from mouse data and an empirical model describing the initial calcium overshoot observed with a glucose step. We first used the model to predict calcium and insulin secretion in some classical *in vitro* studies in mouse islets. We then simulated, after appropriate parameter scaling, several *in vivo* tests in humans, describing the experimental data accurately, in subjects with both normal glucose tolerance and T2D. We also described the well-known enhancement of first-phase insulin secretion with insulin resistance and its reduction with fasting hyperglycemia. Based on a variety of simulations, we proposed that the calcium-independent amplifying pathway is the key defect in T2D, while the loss of first-phase secretion is mainly a consequence of an altered equilibrium of the immediately releasable pool. Another interesting finding is that an accurate description of the experimental data, both *in vitro* and *in vivo*, can be obtained without complex descriptions of the granule status.

Pedersen et al. (2019) investigated how calcium and the granule pools interact to control dynamic insulin secretion, by comparing simulations with three model variants to experimental data in mouse islets. Experimental protocols included different glucose steps, a staircase glucose test, intermittent tolbutamide injection at low glucose and glucose pulses, with measurement of insulin secretion and cytosolic calcium. The first basic model includes a readily releasable pool of granules, and insulin secretion is a function of its size and cytosolic calcium. The pool is replenished by a glucose dependent refilling, described with an empirical function. The second model includes also a pool of docked granules, with a glucose dependent priming rate (transfer from the docked to the readily releasable pool). Glucose dependent refilling takes place in the docked pool. The third model includes an additional glucose-independent refilling of the readily releasable pool, which, affecting the pool size, contributes to insulin secretion. As the third model reproduces the data most accurately, and the staircase experiment in particular, the authors conclude that both calcium and the pool size contribute to shaping insulin release, without the need to assume heterogeneity of the readily releasable pool. The study briefly discusses the possible mechanisms underlying β-cell dysfunction in T2D, without simulations, hypothesizing a defect in both refilling and calcium signaling, in contrast to what proposed in Grespan et al. (2018).

The models illustrated above share several principles, such as the existence of one or more granule pools underlying first-phase insulin secretion and of pool refilling, which ensures granule resupply and determines sustained insulin secretion. However, the description of the mechanisms by which glucose controls granule trafficking often differs remarkably. For instance, not all models represent calcium explicitly, or include a complete representation of the glucose concentration-insulin secretion dynamic relationship. The level of detail of the representation of the known processes is also variable, and some models attempt to describe more precisely the β-cell physical structure, while others have a more abstract approach. The demonstration of the ability of the model to describe experimental data accurately differs widely and only some models show direct comparisons with real data. To our knowledge, only our study supports the hypotheses concerning the defects in T2D showing that the model correctly reproduces the experimental data in these patients.

#### Incretin Effect

The effects of the incretin hormones GIP and GLP-1 on insulin secretion have been often empirically described in some models of glucose homeostasis simulating an OGTT or a meal test, as discussed in the dedicated section below. In contrast, few models have specifically represented the incretin effect to describe experimental data, in spite of its considerable importance.

As concerns the *in vivo* models, the model by Overgaard et al. (2006), described above, represented the incretin effect as an additive term on sustained insulin secretion, using the OGTT glucose concentration increment over baseline as a surrogate of incretin hormone concentrations, which were not measured. The model also included a more complex effect on early secretion. C-peptide was not available to employ the standard insulin secretion method and the model was used to fit insulin concentration during an IVGTT and an OGTT. However, the parameter estimation method treated the IVGTT and OGTT data as if they were from different subjects, as the purpose was to compare the β-cell function parameters rather than to assess the incretin effect. The validity of the incretin effect estimate is thus not based on solid grounds and is untested.

The models by Dalla Man et al. (2010, 2016) are elaborations from the classical insulin secretion models of the group, in which the action of GLP-1 was described as a multiplicative factor on incremental insulin secretion. Four models were proposed to express the dependence of this factor on GLP-1 concentration during a hyperglycemic clamp with concomitant GLP-1 infusion, concluding that a linear dependence on GLP-1 concentration and its derivative was the most appropriate model (Dalla Man et al., 2010). The model variant applied to a meal test (Dalla Man et al., 2016) used a simple linear relationship with GLP-1, but neglected the contribution of GIP.

The model by Tura et al. (2014), an extension of our previous OGTT model (Mari et al., 2002), was conceived to interpret the classical test for the assessment of the incretin effect based on an OGTT and an intravenous glucose infusion matching the OGTT glucose levels. In this model, incretins were assumed to affect both early insulin secretion (increasing the early secretion parameter of the OGTT model) and sustained insulin secretion, by means of a time-dependent incretin potentiation factor. Incretin potentiation was determined from the simultaneous analysis of the OGTT and the intravenous test, without assuming a specific dependence on incretin hormones. The model was applied to various studies, in particular to quantify the sensitivity to incretins in normal and T2D subjects, where an approximate linear relationship between incretin concentration and potentiation was found (Tura et al., 2017).

*In vitro*, we are not aware of models in this area, except for our recent study (Grespan et al., 2020). This model is an extension of our previous β-cell model (Grespan et al., 2018) and describes a transient effect of GIP and GLP-1 on calcium and a sustained effect on the refilling of the immediately releasable pool. Because of the lack of relevant *in vitro* data, the model has been tested on a variety of *in vivo* studies using incretin infusions or an OGTT. The model has revealed an unexpected saturative action of GIP.

#### β-Cell Function Deterioration

Progressive β-cell function loss is a hallmark of the transition to T2D and diabetes progression, but the mechanisms responsible of this phenomenon are still poorly understood. An early attempt to describe this phenomenon was made by Topp et al. (2000), who proposed a simple glucose-insulin homeostatic model in which β-cell mass was dynamically regulated by glucose levels. While moderate hyperglycemia, e.g., originating from insulin resistance, induced an increase in β-cell mass and insulin secretion, marked hyperglycemia produced β-cell mass reduction due to glucose-induced acceleration of β-cell death. The mathematical formulation of this process led to the interesting finding that the system had a stable equilibrium point, where glucose was maintained in presence of moderate disturbances in insulin resistance, but also an unstable point, beyond which glucose control was lost and β-cell mass was progressively reduced to zero. This model not only represented the known inverse relationship between insulin sensitivity and secretion, but also offered a possible explanation of the transition toward diabetes, that successive studies showed to occur in a short time window (e.g., Ferrannini et al., 2004; Tabak et al., 2009). Interestingly, the success of the model has been greater than its adherence to experimental data. The study was in fact based on simulations only, there is no evidence of such dramatic changes in β-cell mass, and the presence of a transition point in the β-cell response, as opposed to a more continue derangement, has not been clearly established.

De Gaetano et al. (2008) followed a similar approach and reached similar conclusions (in particular on the equilibrium points), but using a more elaborate model in the attempt to reconcile the mathematical description with the physiological knowledge, thus permitting a more direct, though limited, evaluation on experimental data (Hardy et al., 2012).

De Gaetano’s model has been recently extended to simulate both short-term glucose perturbations and longitudinal long-term changes in insulin sensitivity and β-cell function (De Gaetano and Hardy, 2019). The model parameters were tuned to simulate longitudinal experimental data from an OGTT (fasting and 30-min glucose and insulin and 2-h glucose), performed sequentially over 4 years in a study including placebo, intensive life style, and metformin treatment. The model has been used to simulate longitudinal changes in insulin sensitivity from the euglycemic clamp, and lifetime changes in OGTT glucose and insulin concentrations and in glycated hemoglobin.

Ha et al. (2016) proposed a different model of the β-cell adaptation to insulin resistance, involving an interplay between changes in β-cell function and mass, which was absent in the previous models. For β-cell mass dynamics, a key factor was the distinction between a glucose-dependent “β-cell metabolic rate” (M), associated with β-cell loss, and an insulin secretion-dependent factor, stimulating β-cell growth. With normal compensation for insulin resistance, the model predicts a rapid increase in β-cell function, followed by a slower mass increase. This combination drives glucose, and thus M, back to normal levels. The increase in mass sustains larger total insulin secretion, while maintaining secretion per β cell normal, a normal M and thus a constant β-cell mass. With a large increase in insulin resistance, the postulated increase in β-cell function is not able to restore glucose, thus leaving M high, a high rate of β-cell loss, leading to uncontrolled glucose and producing massive β-cell mass loss, as in Topp’s model. As previous models, Ha’s model is characterized by two equilibrium points (normal and diabetic condition).

Ha’s model has been recently expanded (Ha and Sherman, 2020) to enrich the glucose homeostasis model, including a dynamic β-cell response [based on Chen et al. (2008)]. The model describes more precisely glucose and insulin concentration obtained from experimental studies, including the OGTT and the IVGTT, allowing prediction of fasting and 2-h glucose after an OGTT both at a given time point and longitudinally, similarly to the model by De Gaetano and Hardy (2019). Several scenarios for T2D onset are simulated, with results compatible with the observations of the longitudinal studies.

In spite of the conceptual interest, demonstrated by the considerable number of citations of Topp’s article, these models remain rather speculative due to the weak relationships with experimental data. The models also typically embed assumptions that do not fully correspond to what is experimentally known (such as the degree of β-cell mass loss in T2D), and the implications of these assumptions on the model results are difficult to predict.

### Insulin Clearance

Insulin kinetics are the intermediate step between the secretion of insulin and its action on glucose fluxes, as for a given insulin secretion response following a glucose stimulus insulin concentration is determined by insulin distribution and clearance. Insulin kinetics have been often considered of secondary importance for glucose homeostasis, as insulin clearance does not appear to be a highly regulated process, as it happens for glucose clearance and insulin secretion (Ferrannini and Cobelli, 1987a). Nevertheless, modeling of insulin kinetics has a time-honored tradition and relevant applications.

Modeling had to face the complexity of the insulin system, in which the endogenous source of insulin is in the portal vein. The liver removes a large fraction of insulin at each transit (∼50%), and thus systemic insulin appearance (or post-hepatic insulin delivery) is only about a half of insulin secretion. In addition, hepatic insulin fractional extraction depends on the insulin levels (Ferrannini and Cobelli, 1987b). Hepatic insulin extraction is thus a potentially crucial determinant of peripheral insulin levels, affecting both first-pass extraction of newly secreted insulin and whole body insulin clearance, through continuous recirculation. However, access to the liver vessels is hardly possible and determination of hepatic insulin extraction from simpler experiments is subject to assumptions. These difficulties are reflected in the historical development of insulin kinetics models.

The early study by Sherwin et al. (1974) proposed a three-compartment representation of insulin kinetics in humans with linear elimination. The model was based on experiments with porcine insulin administered in a peripheral vein as bolus injection and primed continuous infusion at different doses, and showed that insulin in a peripheral compartment, rather than in plasma, was a more direct determinant of glucose utilization. This concept was later used in the glucose utilization model discussed above (Insel et al., 1975).

In early investigation, the linearity of insulin kinetics was debated. Tiran et al. (1979), based on experiments in dogs with both peripheral and portal insulin infusion, developed a circulatory model describing multiple organs, and recognized that a non-linear insulin elimination component was necessary to describe the data consistently. Non-linearity was, however, attributed to peripheral insulin removal, rather than the liver.

Saturation of insulin clearance in humans, as potentially related to insulin binding to receptors, was reported by Jones et al. (1984), who proposed a simple insulin kinetics model with single-compartment insulin distribution and receptor-mediated and non-receptor-mediated insulin degradation. The inclusion of receptor-mediated insulin degradation made the model non-linear. The model was able to describe data from a stepped insulin infusion experiment, causing an increase in peripheral insulin concentration up to ∼1 nmol/L.

Thorsteinsson and colleagues reaffirmed in various studies [summarized in Thorsteinsson (1990)] the importance of including saturation of insulin clearance, in order to explain steady state insulin data after constant infusion of porcine insulin at different rates (insulin concentration up to ∼6 nmol/L), with clamped glucose. Their model, which included a simple Michaelis–Menten function of insulin concentration to quantify insulin removal, accurately described the data in normal and type 1 diabetic subjects. Both Jones’ and Thorsteinsson’s models required assumptions on post-hepatic insulin delivery from endogenous insulin secretion: Thorsteinsson (1990) assumed a constant rate [as in Sherwin et al. (1974)], as C-peptide was substantially unchanged during insulin infusions, while Jones et al. (1984) assumed an exponential decrease, with time constant obtained from previous studies.

Hovorka et al. (1993) extended Jones’s model, describing insulin distribution with three compartments (representing plasma, hepatic, and interstitial insulin), insulin binding to the hepatic and peripheral receptors and receptor-mediated and non-receptor-mediated insulin degradation. The model adequately described data from euglycemic clamps with peripheral insulin infusions at two doses, causing an increase in peripheral insulin concentration up to ∼12 nmol/L. Insulin secretion during the clamps, calculated from C-peptide data, was also accounted for in the analysis.

A more sophisticated multiscale model was developed later in rats by Koschorreck and Gilles (2008), representing the processes described in Hovorka’s model and adding the kinetics of phosphorylation and internalization of the insulin hepatic receptors. The model was able to reproduce experimental data from the literature using parameter values taken from *in vitro* experiments and previous studies, without adjustment. However, several assumptions were required to define the complex structure of the model.

While the approaches discussed above were based on exogenous insulin infusion, the possibility to assess insulin secretion from C-peptide, discussed above, offered a new way to estimate insulin clearance and in particular hepatic insulin extraction. The pioneering study by Eaton et al. (1983) employed the previously described C-peptide model (Eaton et al., 1980) and a three-compartment insulin model representing hepatic, vascular, and extravascular spaces, derived from Sherwin’s study (Sherwin et al., 1974). A key assumption was the use of fixed parameter values for the insulin kinetics model, determined from previous experiments, except for insulin removal from the hepatic compartment and the transfer rate from liver to plasma. This assumption was crucial to allow estimation of hepatic insulin extraction from tests without exogenous insulin infusion. The analysis of OGTT’s at increasing doses revealed decreasing insulin extraction by the liver in the presence of increasing insulin exposure. The dependence of hepatic insulin extraction from insulin levels was, however, not modeled.

Other studies followed Eaton’s approach, using measurement of insulin and C-peptide without exogenous insulin infusion, to quantify insulin clearance or hepatic extraction. Cobelli and Pacini (1988) employed a two-compartment C-peptide kinetics model and a single-compartment insulin model to describe data from an IVGTT. The single-compartment insulin model described the fate of post-hepatic insulin delivery. Insulin secretion from C-peptide and post-hepatic insulin delivery from insulin were determined, together with the parameters of the two kinetic models, assuming specific relationships with glucose concentration, similarly to a previous study (Toffolo et al., 1980). Hepatic insulin extraction was calculated as the relative difference between insulin secretion and post-hepatic insulin delivery. Thus, this model used specific relationships between glucose concentration and insulin secretion or post-hepatic insulin delivery to resolve the indeterminacy that Eaton et al. (1980) overcame with assumptions on the insulin kinetics model. The approach estimates insulin secretion and post-hepatic insulin delivery simultaneously with insulin and C-peptide kinetics parameters. As insulin secretion depends on C-peptide kinetics and post-hepatic insulin delivery on insulin kinetics, the concomitant estimation of the parameters of these processes may lead to an inaccurate separation of their role, resulting in biased parameter estimates. In addition, the insulin kinetics model assumes a constant clearance, which contradicts the finding of a variable hepatic insulin extraction, as physiologically hepatic insulin extraction contributes to whole-body insulin clearance.

Measurement of insulin and C-peptide without exogenous insulin infusion was the strategy adopted also by Thomaseth et al. (1996), who modeled OGTT data with a single-compartment insulin model as in Cobelli and Pacini (1988), but assuming constant hepatic insulin fractional extraction. Tura et al. (2001), using this model, showed reasonable concordance between the model estimates of hepatic extraction and the results of *trans*-hepatic catheterization. Piccinini et al. (2016), to describe mixed meal test data, proposed a three-compartment insulin kinetics model, with insulin cleared linearly from the central compartment and non-linearly from a peripheral compartment, assumed to represent the liver and to receive secreted insulin. The non-linear clearance component was described as a linear function of plasma glucose concentration, with negative slope, an assumption with uncertain physiological basis. To determine the model parameters from the data, specific relationships between glucose concentration and insulin secretion were assumed, similarly to Cobelli and Pacini (1988). Assumptions on the insulin kinetics model were also necessary, similarly to Eaton et al. (1983), but in this case a model for hepatic insulin extraction was used.

The models following Eaton’s approach described above relied on experiments in which only endogenous insulin secretion was present. In this condition, estimates of insulin clearance and hepatic insulin extraction are crucially dependent on the model assumptions, as in Eaton’s model. A more robust approach was used by Toffolo et al. (2006), Campioni et al. (2009), and Polidori et al. (2016), who relied on the so-called insulin-modified IVGTT, in which insulin is intravenously infused over 5 min, 20 min after the glucose bolus. The advantage of this experiment is the presence of both an endogenous and an exogenous source of insulin appearance, which allows clearer separation of hepatic and extrahepatic insulin clearance. Toffolo et al. (2006) used the insulin kinetics model developed by Cobelli and Pacini (1988). Their analysis was further developed by Campioni et al. (2009), who used a piecewise linear description of hepatic fractional extraction.

The model developed by Polidori et al. (2016) represented insulin kinetics with a single-compartment, in which the influx is the sum of insulin infusion and post-hepatic insulin appearance, i.e., insulin secretion times one minus the hepatic fractional extraction, and the outflux is the product of insulin concentration and insulin clearance. Insulin clearance includes a hepatic component (hepatic fractional extraction times hepatic plasma flow) and an extrahepatic component (a constant). Individual hepatic insulin extraction is described with a linear or a Michaelis–Menten function of insulin delivery to the liver (estimated from insulin concentration and secretion), depending on the subject’s data. While the model provides a clinically interesting method, indeed used in successive studies (e.g., Smith et al., 2020), it is based on a single-compartment simplification of insulin kinetics and it uses an ambiguous (as subject-dependent) representation of the saturative hepatic insulin extraction.

Although the historical concept that insulin clearance is not substantially regulated has not been superseded, recent studies, often based on modeling, have emphasized that insulin clearance is modulated by several factors and that this modulation affects insulin levels and therefore glucose homeostasis (e.g., Bojsen-Møller et al., 2018; Smith et al., 2020). As insulin levels influence insulin clearance due to saturation of insulin removal, the study of these factors requires a precise quantification of insulin clearance independent of the prevailing insulin levels. For instance, it is known since long time that insulin clearance is inversely related to insulin resistance (Haffner et al., 1992). However, since insulin resistance also produces relative hypersecretion and thus hyperinsulinemia, distinguishing the direct influence of insulin resistance on insulin clearance from that mediated by the saturative effects of hyperinsulinemia is challenging. This is an area where modeling is particularly relevant and progress above the current models is needed. One major drawback of most of the models discussed here is in fact the use of compartments, which distorts the relationships between the mathematical representation and the physiological system, with unpredictable consequences on the interpretation of the estimates of insulin clearance and hepatic insulin extraction. Circulatory models, as the historical Tiran’s model (Tiran et al., 1979), offer a more appropriate solution. Furthermore, models should embed a clear representation of saturation phenomena and provide parameters quantifying insulin clearance or hepatic insulin extraction that do not depend on insulin levels. Experimental data should also include both exogenous and endogenous sources of insulin delivery, to reduce the impact of model assumptions. We have recently proposed a model inspired on these principles (Bizzotto et al., 2018), and the study is still underway.

### Glucose Homeostasis

As anticipated in the Section “Introduction,” models of glucose homeostasis have a long history. The direct ancestor of the most famous HOMA model (Turner et al., 1979) has been one of the earliest attempts to develop a glucose homeostasis model based on a variety of data, but the study follows a logic that is rather far from the current conception of glucose homeostasis models. Perhaps the earliest ambitious attempt to develop a comprehensive model is that by Guyton et al. (1978) at Joslin and MIT, who used the state of the art knowledge of those times for representing glucose metabolism and insulin secretion. Since those early attempts, many glucose homeostasis models with various aims and degree of complexity have been proposed (see e.g., Ajmera et al., 2013). The evolution of these models to some extent has followed the increase in detailed knowledge of the components of the glucose homeostatic system. Here we review some of the more recent models in this field, highlighting the different aims and degree of adherence to physiological knowledge.

Two widely cited models of glucose homeostasis were developed in Cambridge (Hovorka et al., 2004) and Padua (Dalla Man et al., 2007). Their success mostly relates to their use for testing the performance of algorithms employed in the so-called artificial pancreas, a closed-loop glucose controller for type 1 diabetes management. The two models were developed based on the consolidated experience of the respective research groups on the use of models in clinical investigation, e.g., in the study of glucose kinetics and insulin action with multiple glucose tracers.

The model of Hovorka et al. (2004) is a compartmental model of the glucose-insulin system, with two compartments for glucose kinetics, one compartment for insulin kinetics (assumed linear), and three distinct delay compartments for the actions of insulin on glucose distribution, utilization and production. Insulin-independent glucose utilization is reduced when glucose concentration is low. Renal glucose excretion is represented as a threshold function of glucose. Two-compartment chains were used to model glucose absorption from the gut and absorption of subcutaneously administered short-acting insulin. Insulin secretion was not represented, as the model was intended to simulate the type 1 diabetes condition. The model was developed based on a previous model of glucose transport and utilization, built on data from an IVGTT with multiple tracers (Hovorka et al., 2002). The model was later extended to simulate glucose regulation in the critically ill (Hovorka et al., 2008), including an insulin secretion component [derived from Hovorka et al. (1998), with the addition of an inhibitory effect of exogenous insulin] and an insulin kinetic component with saturable removal. The glucose kinetic model was modified to account for saturable effects of insulin concentration on glucose transport and utilization. Insulin sensitivity was also assumed time-dependent, a necessary feature in the critically ill.

In the model of Dalla Man et al. (2007), glucose and insulin kinetics are represented with two-compartment submodels and glucose absorption is modeled via compartments associated to the stomach and gut according to a previous study (Dalla Man et al., 2006). The insulin kinetics model accounts for non-constant hepatic extraction. The action of insulin on glucose utilization and production is represented by non-linear functions of delayed insulin (obtained from a chain two-compartment model). Renal glucose excretion is represented as a threshold function of glucose. Insulin secretion is described by the model of Breda et al. (2001), discussed above. This model was successively expanded to describe other aspects of glucose homeostasis, including: a more detailed representation of the dependence of glucose utilization on glucose concentration, glucagon secretion and kinetics and its effects on glucose production, in type 1 diabetic patients (Dalla Man et al., 2014); circadian variations in insulin sensitivity [derived from Visentin et al. (2015)]; the “dawn” phenomenon (early-morning increase in blood glucose concentration) (Visentin et al., 2018); and a three-compartment model of insulin kinetics (Visentin et al., 2020). The incretin effect was not modeled. A computer simulator of single meal scenarios based on the 2007 model has been accepted by the Food and Drug Administration as a substitute to animal trials for the preclinical testing of control algorithms in artificial pancreas studies.

Another model of glucose homeostasis oriented toward the artificial pancreas application is that by Fabietti et al. (2006). A glucose-insulin model derived from the minimal model was expanded to include a component describing glucose absorption from meals as a function of meal carbohydrate composition. Circadian variations in insulin sensitivity were also represented. The model was tested on a limited data set in type 1 diabetic subjects. The model by Brubaker et al. (2007), developed on OGTT data, was devised to illustrate the importance of the incretin effect and, more generally, to simulate glucose dynamics under various conditions, some of which compared with published data. The foundations and testing of these models are somewhat weaker than those of the models discussed above.

In the PKPD field, several compartmental models have been proposed to describe glucose homeostasis, with progressive improvements allowed by the available data, resulting in an increased level of completeness. The first models were built on different studies analyzed together, in which glucose was given intravenously (Silber et al., 2007) or both intravenously and orally (Jauslin et al., 2007), following various protocols and using glucose tracers. The models included compartments to represent glucose and insulin kinetics and oral glucose absorption, with delayed glucose and insulin signals affecting glucose clearance and production, following a logic similar to that of the models described above. Simple models were used for insulin secretion and its increase after glucose ingestion; in the model on intravenous glucose data, first-phase insulin secretion was represented only in healthy subjects and omitted in T2D patients. Extensions of these models included the description of circadian rhythms [based on 24-h glucose and insulin profiles, (Jauslin et al., 2011)], glucagon kinetics and glucose production dependence from glucose, insulin and glucagon [using meal tests with glucagon measurement, (Schneck et al., 2013)], gastric emptying and glucose absorption [using paracetamol, (Alskär et al., 2016)], and scaling factors to account for interspecies differences (Alskär et al., 2017). These models employ empirical descriptions of their components (e.g., insulin secretion), sufficient to predict the available data adequately. On the other hand, the population parameter estimation methods, typical of PKPD and used with these models, quantify inter-individual variability in the model parameters; these results are reported in the publications and are a potential advantage for realistic simulations of clinical studies. To the best of our knowledge, such estimates have not been reported for most of the other models.

The models described above have a variable degree of complexity, but mathematical representations are kept parsimonious, aiming at describing only mechanisms that are essential to explain adequately the observed data. A different approach is taken by two significant models that represent the details of the glucose homeostatic system up to the cell level. One aim of this approach is the possibility to predict the clinical effects of drugs, based on the knowledge of their molecular mechanisms.

The model of Schaller et al. (2013) follows the principles of systems biology and pharmacology (multiscale, or hierarchical, approach), with integration of multiscale data from the subcellular level (e.g., receptor-mediated action and clearance of insulin) to the whole-body level (e.g., distribution of blood flow across the organs). The model considers kinetics models for glucose, insulin, and glucagon. The organs relevant for each of these components are described in detail and represented in their interconnection through the circulation, based on an approach known as physiology-based pharmacokinetics modeling (Mould and Upton, 2012). The parameter values were taken from the literature, and the values of the most influential parameters were adjusted to fit several standard *in vivo* tolerance tests. The ability of the model to represent the behavior of the subsystems appropriately was, however, not tested in detail.

The multiscale approach by Nyman et al. (2011) used the homeostasis model of Dalla Man et al. (2007) as a general framework, and expanded the submodel describing glucose utilization in insulin-dependent tissues by including an adipose tissue module. The adipose tissue module described glucose uptake via the translocation of the GLUT4 transporter, enhanced by insulin signaling, which was represented including a feedback model of internalization of the insulin-receptor complex. The model reproduced some *in vitro* and *in vivo* data. This framework was specifically developed to elucidate the link between *in vitro* insulin signaling and *in vivo* glucose homeostasis, which is of interest in the study of insulin resistance. On the other hand, the usefulness of the whole multiscale model for simulation of glucose homeostasis was not demonstrated.

Accurate representation of glucose homeostasis is difficult because of the complexity of the physiological system and its incomplete knowledge. All models embed approximations, the influence of which on the accuracy of the simulations is hard to predict. This difficulty is illustrated in one of our studies discussed above (Bizzotto et al., 2016), in which we present a glucose kinetics model that explains the dependence of glucose clearance on glucose concentration, a phenomenon represented only in some models. In this study, we describe a partial glucose homeostasis model and show that neglecting this phenomenon leads to relevant differences in the predicted glucose concentration during an OGTT or an insulin infusion in T2D subjects. Therefore, performing a critical evaluation or assessing the domain of validity of glucose homeostasis models is practically impossible in general. The model complexity is not necessarily an advantage in itself; a better performance is more likely to be obtained from the models that embed components separately developed in specific studies and are tested on significant clinical datasets.

## Conclusion

Although this review covers only some topics, it highlights the usefulness and vitality of modeling in the area of glucose homeostasis. Models for clinical use appear to take the lion’s share for understandable reasons, but we have underlined the interest of models that provide explanations of the possible mechanisms underlying complex phenomena, such as insulin secretion. There is obviously large space for new models, as several aspects of glucose homeostasis are still underexplored. In a review of almost 20 years ago (Mari, 2002), we identified modeling of the OGTT as a promising area, particularly for insulin secretion. As this has now come true, we propose that two broad areas might be new challenges for the future: the use of glucose homeostasis models to gain deeper insight in the progression toward T2D, which involves multiple interacting factors with a still unclear quantitative role; and the use of models to link *in vitro* and *in vivo* findings, for which interesting efforts have been recently made for insulin secretion.

## Author Contributions

AM planned the manuscript and wrote most of it. RB, EG, and AT wrote specific sections. All authors critically reviewed the manuscript.

## Conflict of Interest

The authors declare that the research was conducted in the absence of any commercial or financial relationships that could be construed as a potential conflict of interest. The reviewer AD declared a shared affiliation with the authors to the handling editor at the time of review.
